# Case Report: dupilumab for severe pruritus in pediatric recessive dystrophic epidermolysis bullosa—a longitudinal clinical assessment and literature review

**DOI:** 10.3389/fimmu.2026.1956336

**Published:** 2026-09-24

**Authors:** Linli Liu, Qianying Li, Yan Liu, Kehan Li, Chunshui Yu

**Affiliations:** Department of Dermatology, Suining Central Hospital, Suining, China

**Keywords:** *COL7A1*, dupilumab, pediatric dermatology, pruritus, recessive dystrophic epidermolysis bullosa

## Abstract

Recessive dystrophic epidermolysis bullosa (RDEB) results from biallelic *COL7A1* variants and causes trauma-induced blistering, scarring, and progressive extracutaneous complications. Severe pruritus may intensify skin injury through an itch-scratch cycle. We report a 7-year-old, 25-kg girl with infantile-onset generalized blistering, severe pruritus, mucosal and nail involvement, scarring, and digital contractures. Histopathology showed a subepidermal blister. Sequencing identified heterozygous *COL7A1* c.5605G>C (p.Gly1869Arg) and c.5532+5G>A, inherited from different parents and therefore occurring in trans. Contemporary ACMG/AMP reassessment classified c.5532+5G>A as pathogenic and p.Gly1869Arg as likely pathogenic; together with the phenotype and segregation findings, these variants supported a molecular diagnosis of RDEB. Because severe pruritus and inflammatory erosions persisted despite supportive care, off-label dupilumab was initiated with a 600-mg loading dose followed by 300 mg every 4 weeks. Routine wound care was continued, while caregiver-reported use of cetirizine drops and topical fusidic acid was intermittent during follow-up. Prospective assessments at baseline and weeks 4, 8, 12, and 16 documented a reduction in pruritus numerical rating scale (NRS) score from 7 to 1 as the primary clinical observation. Secondary descriptive changes included decreases in the Epidermolysis Bullosa Disease Activity and Scarring Index (EBDASI) activity score from 52 to 32 and pain NRS from 4 to 1, and a change in hand-function grade from 3 to 2. Photographs showed fewer active erosions and crusted lesions, whereas established scarring and contractures persisted. No adverse events considered related to dupilumab were observed during 16 weeks. Because this was an uncontrolled observation with continued background care, the changes do not establish causality. No patient-specific type 2 inflammatory biomarkers were measured.

## Introduction

Recessive dystrophic epidermolysis bullosa (RDEB) is an inherited skin-fragility disorder caused by biallelic pathogenic variants in *COL7A1*. Deficient or dysfunctional type VII collagen weakens anchoring fibrils at the dermal-epidermal junction, allowing minor trauma to cause blistering and erosions that heal with scarring. Recurrent injury may lead to chronic wounds, nail dystrophy, mucosal involvement, contractures, infection, anemia, and impaired quality of life (1, 2). Pruritus is common and difficult to treat. In the prospective PEBLES registry, recent itch was reported at 93% of 243 assessments involving 50 individuals with RDEB (3). Scratching adds mechanical trauma to fragile skin and may perpetuate an itch-scratch cycle.

Immune profiling of 84 patients with RDEB identified a type 2-skewed signature in moderate-to-severe disease at the cohort level and highlighted the interleukin (IL)-4/IL-13 pathway as a potential therapeutic target (4). Experimental studies have also shown that IL-4 and IL-13 can activate sensory neurons through IL-4 receptor alpha-dependent signaling and promote chronic itch (5). These findings provide a rationale for IL-4/IL-13 blockade but cannot assign a type 2 inflammatory endotype to an individual patient without biomarker evidence. Initial reports of off-label dupilumab in RDEB focused mainly on RDEB pruriginosa (6, 7); later small series and retrospective cohorts included severe and intermediate RDEB (8–10). The evidence remains limited by small, clinically heterogeneous samples and inconsistent outcome measures.

We report a 7-year-old girl with infantile-onset generalized RDEB, severe pruritus, widespread inflammatory erosions, scarring, and digital contractures. Her response to 16 weeks of off-label dupilumab was assessed prospectively at five time points using the EBDASI activity score, pruritus and pain numerical rating scales, hand-function grading, and paired photographs. This case adds to the limited clinical experience with dupilumab in children with generalized RDEB and severe pruritus.

## Case description

### Clinical presentation

A 7-year-old girl was referred with recurrent generalized blistering and severe pruritus for more than 6 years. The eruption began in infancy with erythematous patches and variably sized vesicles and bullae on the trunk and extremities. The blisters ruptured readily and recurred despite intermittent symptomatic treatment at local clinics. Oral antihistamines and topical fusidic acid provided only transient relief. Hereditary epidermolysis bullosa had been suspected, but no definitive diagnostic assessment had been completed. There was no known family history of a similar blistering disorder.

Examination showed sparse scalp hair, pallor, limited tongue protrusion, superficial oral erosions, and partial loss or dystrophy of the fingernails and toenails. Widespread dark-red erythema and lichenification affected the trunk and extremities. Multiple thin-roofed vesicles and bullae were present, some with turbid fluid. Localized bacterial superinfection was clinically suspected in some lesions containing turbid blister fluid and yellow-brown crusting, although microbiological cultures were not performed. Nikolsky’s sign was positive, with epidermal separation induced by lateral mechanical pressure applied to perilesional skin. Excoriated lesions formed bright-red erosions with exudation and yellow-brown to hemorrhagic crusts. The palms showed erosions, scarring, and digital contractures (**Figures 1A–C**).

**Figure 1:**
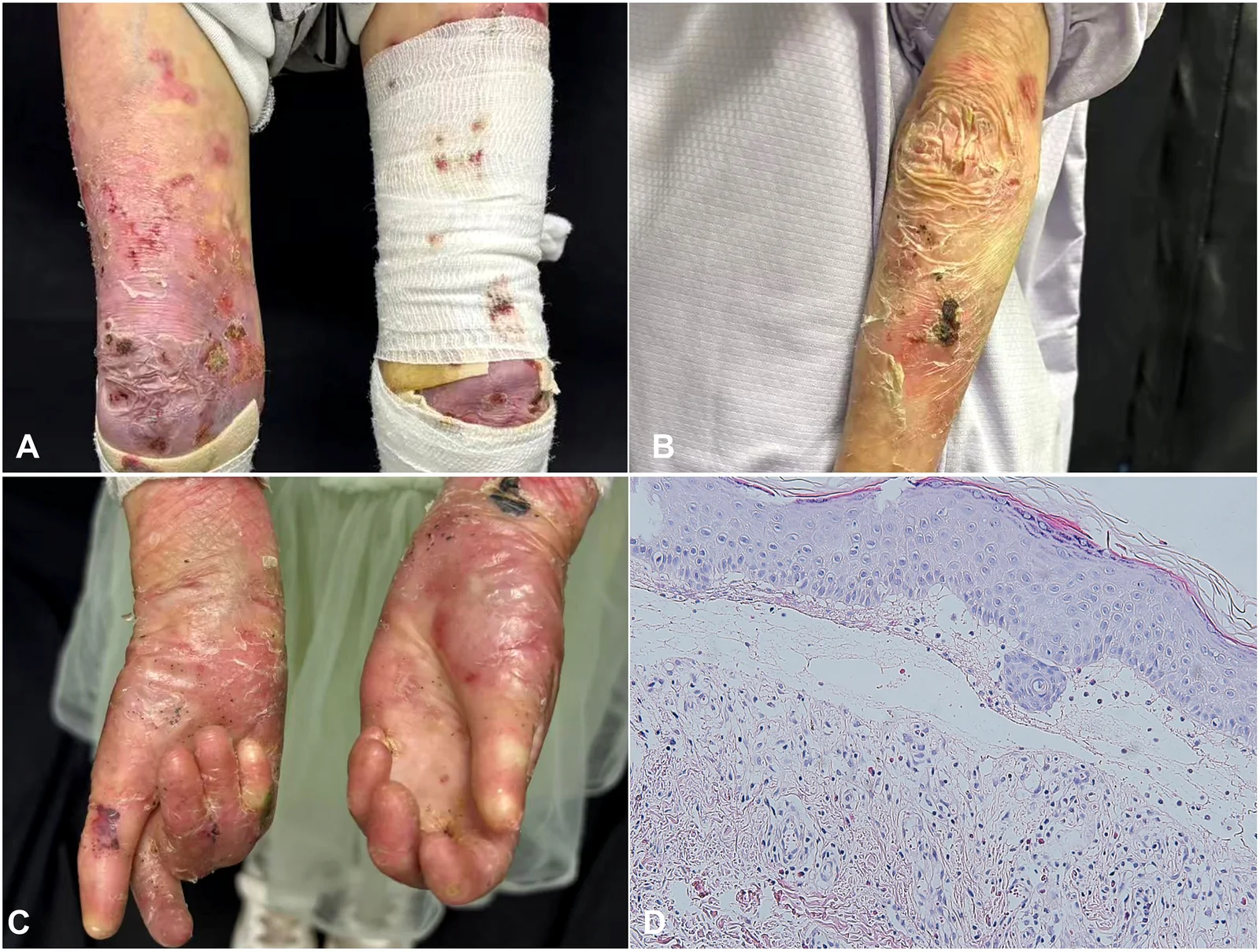
Clinical and histopathological features of recessive dystrophic epidermolysis bullosa at presentation. **(A–C)** Clinical photographs of a 7-year-old girl with recurrent blistering and severe pruritus for more than 6 years. **(A)** Both upper extremities show widespread erythema and lichenification with excoriations, erosions, and yellow-brown to hemorrhagic crusts. **(B)** An affected forearm shows lichenified skin with erosions and crusted lesions. **(C)** Palmar involvement includes erosions, scarring, and digital contractural changes. **(D)** Hematoxylin and eosin staining of a lesional biopsy from the right forearm shows hyperkeratosis and a subepidermal blister containing fibrin and scattered neutrophils. The superficial dermis contains a predominantly lymphocytic perivascular infiltrate with scattered eosinophils, a nonspecific finding that was not interpreted as evidence of type 2 inflammation. Routine hematoxylin and eosin examination was supportive but was not sufficient for precise EB subtype characterization or localization of the cleavage plane. Original magnification, x200.

### Diagnostic assessment

Apart from body weight, which was recorded for dose selection, systematic anthropometric assessment and laboratory evaluation of anemia, protein-energy status, and micronutrient deficiencies were not performed. Histopathological examination of a right-forearm lesion showed hyperkeratosis and a subepidermal blister containing fibrin and scattered neutrophils. The superficial dermis contained a predominantly lymphocytic perivascular infiltrate with scattered eosinophils (**Figure 1D**). These eosinophils were considered nonspecific. Routine histopathology supported the diagnosis of epidermolysis bullosa but was not subtype-specific; type VII collagen immunofluorescence was not performed.

Whole-exome sequencing identified two heterozygous *COL7A1* variants (NM_000094.4): c.5605G>C (p.Gly1869Arg) and c.5532+5G>A. Sanger sequencing showed paternal inheritance of c.5605G>C and maternal inheritance of c.5532+5G>A, confirming that the variants occurred in trans (**Figures 2A, B**). Evidence obtained directly from this patient comprised detection of both variants, parental segregation, confirmed phase, and phenotype-genotype concordance.

**Figure 2:**
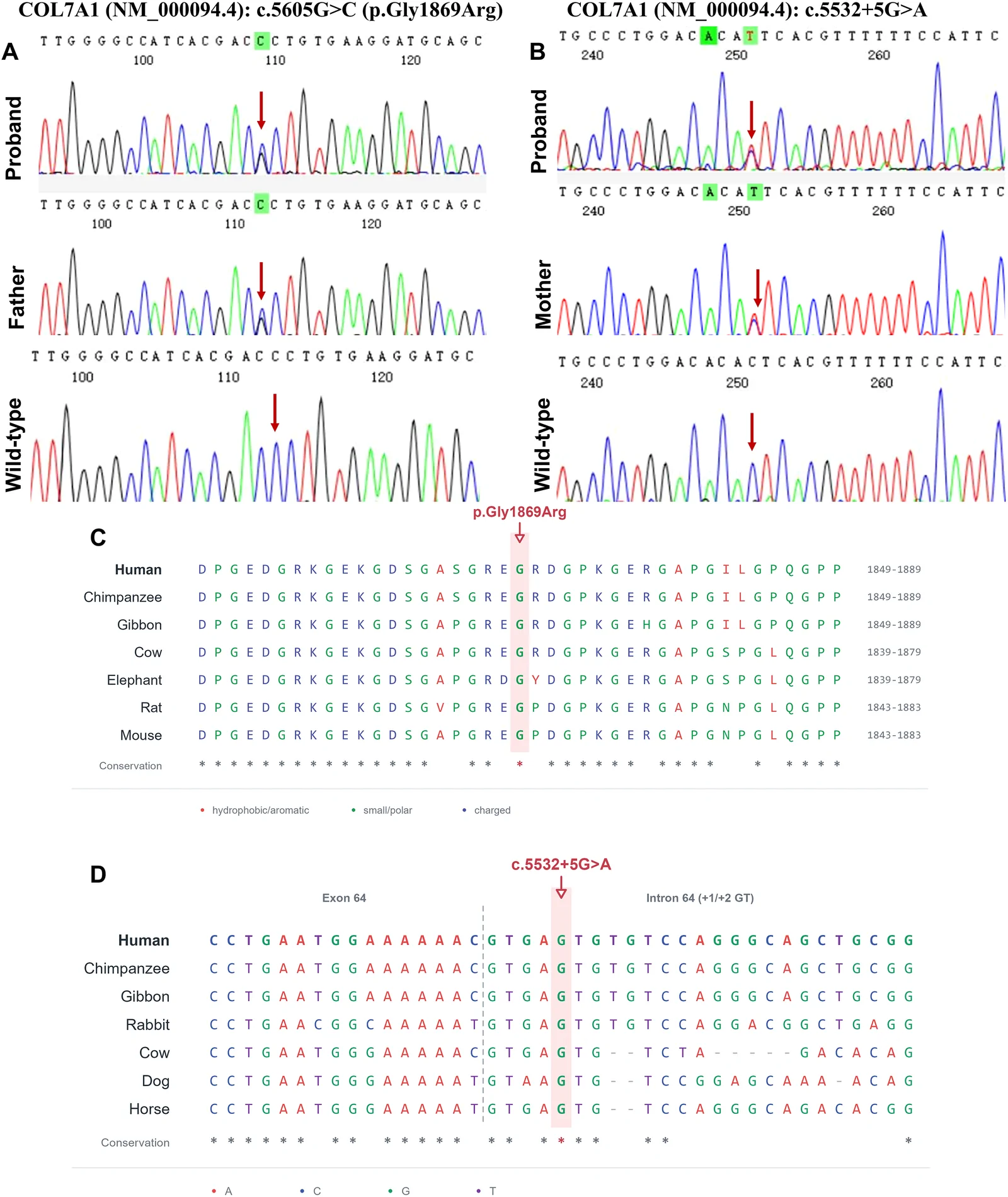
Familial segregation and evolutionary conservation of the COL7A1 variants. **(A)** Sanger sequencing confirms heterozygous COL7A1 c.5605G>C (p.Gly1869Arg) in the proband and her father. The wild-type control trace lacks the substitution. **(B)** Sanger sequencing confirms heterozygous COL7A1 c.5532+5G>A in the proband and her mother. The wild-type control trace lacks the substitution. Red arrows indicate variant positions. Inheritance from different parents supports an in-trans configuration. **(C)** A 41-residue window from full-length mammalian collagen alpha-1(VII) chain orthologues was aligned with Clustal Omega. Gly1869 is invariant across the seven displayed species. The human reference is UniProt Q02388, and p.Gly1869Arg is highlighted. Amino acids are colored by physicochemical class; asterisks mark residues identical across all sequences. **(D)** An Ensembl Compara mammalian genomic alignment shows the relevant exon-intron donor region (NM_000094.4; GRCh38). The +5 guanine affected by c.5532+5G>A is conserved. The dashed line marks the exon-intron boundary, and the canonical +1/+2 GT dinucleotide is indicated. Nucleotides are color-coded; asterisks denote identity across the displayed species.

Under the ACMG/AMP framework and Bayesian point-based calibration (11, 12), c.5532+5G>A met PVS1, PM3_Supporting, and PM2_Supporting and was classified as pathogenic; ClinVar also contains multiple pathogenic submissions without conflicts (16). The splice evidence informing PVS1 and prior in-trans observations informing PM3_Supporting came from published or external cases. In an unrelated patient carrying the same variant, RNA analysis showed complete in-frame exon 64 skipping and skin immunofluorescence showed undetectable type VII collagen at the basement membrane zone (17). Patient-derived RNA was unavailable in the present case; these published findings therefore support variant interpretation but do not establish the transcript or protein consequence in this child.

The p.Gly1869Arg variant met PM1_Supporting, PM3, PM2_Supporting, PP3, and PP4_Supporting (six Bayesian points) and was classified as likely pathogenic. PM1 was limited to supporting strength because the substitution affects the obligatory glycine of a Gly-X-Y repeat within the type VII collagen triple-helical region (residues 1254-2784) (13), where glycine is required for close packing (14). This location-based evidence was distinct from PP3, and no assay established the effect of this specific substitution. PP4 was also limited to supporting strength because the phenotype was consistent with *COL7A1*-related dystrophic epidermolysis bullosa, whereas routine histopathology was not subtype-specific and neither immunofluorescence mapping nor transmission electron microscopy was performed. Cross-species alignments showed conservation of Gly1869 and the intronic +5 guanine (**Figures 2C, D**). No variant-specific functional assay was performed for p.Gly1869Arg.

The pathogenic and likely pathogenic *COL7A1* variants in trans, together with the compatible phenotype and familial segregation, supported a molecular diagnosis of RDEB.

### Therapeutic intervention

Severe pruritus, recurrent blistering, and inflammatory erosions persisted despite supportive care. Off-label dupilumab was initiated when the patient weighed 25 kg. Because no RDEB-specific regimen has been validated, we selected the current US weight-tiered regimen approved for atopic dermatitis in patients aged 6–17 years weighing 15 to <30 kg: a 600-mg loading dose followed by 300 mg every 4 weeks (23). Every-2-week regimens reported in RDEB involved heterogeneous ages, body weights, phenotypes, and follow-up and do not establish a more appropriate schedule for this 25-kg child (**Table 1**). The dose was therefore extrapolated from an established pediatric indication. No validated RDEB-specific dose, treatment duration, or stopping rule is available. Routine wound care continued. According to the caregiver, cetirizine drops and topical fusidic acid were used intermittently and without a fixed schedule during the 16-week follow-up. No prospective medication or application log was maintained. Therefore, the precise frequency and duration of either medication, the extent and sites of fusidic acid application, whether each application was restricted to lesions with suspected superinfection, and changes in use over time could not be reliably determined.

**Table 1:** Published RDEB-specific experience with dupilumab, arranged by phenotype: RDEB pruriginosa reports followed by generalized severe/intermediate or mixed-phenotype RDEB reports and the present case.

Study; phenotype category	n	Age/sex	COL7A1 evidence	Dupilumab regimen; follow-up	RDEB-specific outcomes	Safety
Shehadeh et al., 2020 (6); RDEB-Pr	1	52 y/F	Two heterozygous variants: c.5772A>C and c.6619-2A>T	600-mg loading; 300 mg Q2W; 12 wk	Pruritus VAS 9 to 5; DLQI 23 to 8; EBDASI 59 to 51	No adverse event reported
Zhou et al., 2021 (18); RDEB-Pr, patient 1 only	1	15 y/M	Two heterozygous *COL7A1* variants; second, single-variant participant excluded	600-mg loading; 300 mg Q2W; 22 mo	Pruritus NRS 8 to 4.5 by month 18; improved sleep; plaques flattened	No adverse event reported
Wang et al., 2021 (19); RDEB-Pr	1	10 y/F	c.7247G>A and c.946G>A	600-mg loading; 300 mg Q2W; 7 mo	Pruritus VAS 5.5 to 1.8; DLQI 13 to 4; marked lesion improvement	No adverse event reported
Caroppo et al., 2022 (20); RDEB-Pr	1	43 y/F	c.7344G>A and c.425A>G	600-mg loading; 300 mg Q2W; 18 mo	Pruritus VAS 9 to 3; sustained clinical improvement	No adverse event reported
Wu et al., 2023 (7); RDEB-Pr, patient 1 only	1	13 y/M	c.6770G>A (p.Gly2257Glu) and c.2551C>T (p.Arg851Cys)	600-mg loading; 300 mg Q4W; 20 wk	VAS 7 to 2; CDLQI 28 to 16; EBDASI 68 to 58; fewer recurrent blisters	No adverse event reported
Yu et al., 2023 (21); RDEB-Pr	2	16 y/F; 13 y/M	Both carried c.8234G>A and c.4128delT	600-mg loading; 300 mg Q2W; 16 wk reported	Patient 1: NRS 6.5 to 1 and DLQI 19 to 0; both had substantial flattening of pruriginous lesions	No adverse event reported
Gewert et al., 2024 (8); severe/intermediate RDEB and RDEB-Pr subgroup	4	Pediatric/adult	RDEB diagnoses reported; mixed-cohort molecular details not fully separable	600-mg loading; 300 mg Q2W; individual follow-up 2–12 mo	RDEB cases showed rapid itch reduction and fewer lesions; intermediate RDEB example: VAS 10 to 2 at 5 mo	No RDEB-specific adverse event identifiable
Caroppo et al., 2024 (22); RDEB	1	12 mo/F	c.7795-1G>A; molecularly confirmed RDEB	200-mg loading; 200 mg Q4W; 3 mo	Pruritus NRS 9 to 2; fewer excoriations and blisters	No adverse event reported
Dou et al., 2025 (9); severe/intermediate RDEB and RDEB-Pr subgroup	7 RDEB (study n=8)	5–38 y; subgroup details not fully separable	Molecularly diagnosed DEB; RDEB subgroup included severe, intermediate, and pruriginosa phenotypes	Age/weight-adapted regimens; mean 44.5 wk (range 12-130)	Overall cohort: pruritus NRS decreased by approximately 59% at week 4 and 50% at week 12; BEBS decreased by 22% at week 12; RDEB-only aggregate outcomes not fully separable	No RDEB-specific adverse event identified
Wang et al., 2026 (10); severe/intermediate RDEB and RDEB-Pr subgroup	5	9–17 y	Biallelic/compound-heterozygous *COL7A1* variants reported	Age/weight-adapted regimens; at least 52 wk; selected long-term patients extended to Q4W	Severe/intermediate RDEB: chronic wounds healed and NRS approximately 2–3 by week 52; RDEB-Pr: durable itch/lesion control	One mild, transient conjunctivitis; no serious adverse event
Present case; generalized RDEB with a prominent pruritic-inflammatory phenotype	1	7 y/F; 25 kg	NM_000094.4: c.5605G>C (p.Gly1869Arg), paternal; c.5532+5G>A, maternal	600-mg loading; 300 mg Q4W; 16 wk	EBDASI activity score 52 to 32; pruritus NRS 7 to 1; pain NRS 4 to 1; hand-function grade 3 to 2; fewer new blisters	No adverse events considered related to dupilumab were observed during the 16-week follow-up; treatment ongoing

BEBS, Birmingham Epidermolysis Bullosa Severity score; CDLQI, Children’s Dermatology Life Quality Index; DLQI, Dermatology Life Quality Index; EBDASI, Epidermolysis Bullosa Disease Activity and Scarring Index; F, female; M, male; NRS, numerical rating scale; Q2W, every 2 weeks; Q4W, every 4 weeks; RDEB, recessive dystrophic epidermolysis bullosa; RDEB-Pr, RDEB pruriginosa; VAS, visual analogue scale.

### Follow-up and outcomes

Outcome assessments were performed prospectively at baseline and weeks 4, 8, 12, and 16. The same EBDASI-trained physician investigator assessed the EBDASI activity score at every visit. Pruritus and pain NRS values reflected symptom severity during the preceding 24 hours and were reported jointly by the child and her caregiver. Photographs were obtained under broadly comparable conditions, with efforts to maintain similar distance, lighting, and positioning, but a strictly standardized photography protocol was not used.

At baseline, the EBDASI activity score was 52, pruritus NRS was 7, pain NRS was 4, and the Colville-Terrill hand-function grade was 3. At weeks 4, 8, 12, and 16, EBDASI activity scores were 47, 40, 36, and 32; pruritus NRS scores were 4, 2, 1, and 1; and pain NRS scores were 2, 1, 1, and 1, respectively. Colville-Terrill grades were 3 at week 4 and 2 at weeks 8, 12, and 16. From baseline to week 16, the EBDASI activity score decreased by 20 points (38%), pruritus NRS by 6 points (86%), and pain NRS by 3 points (75%); hand-function grade changed from 3 to 2 (**Figure 3I**). The family reported fewer new blisters. No adverse events considered related to dupilumab were observed during the 16-week follow-up.

**Figure 3:**
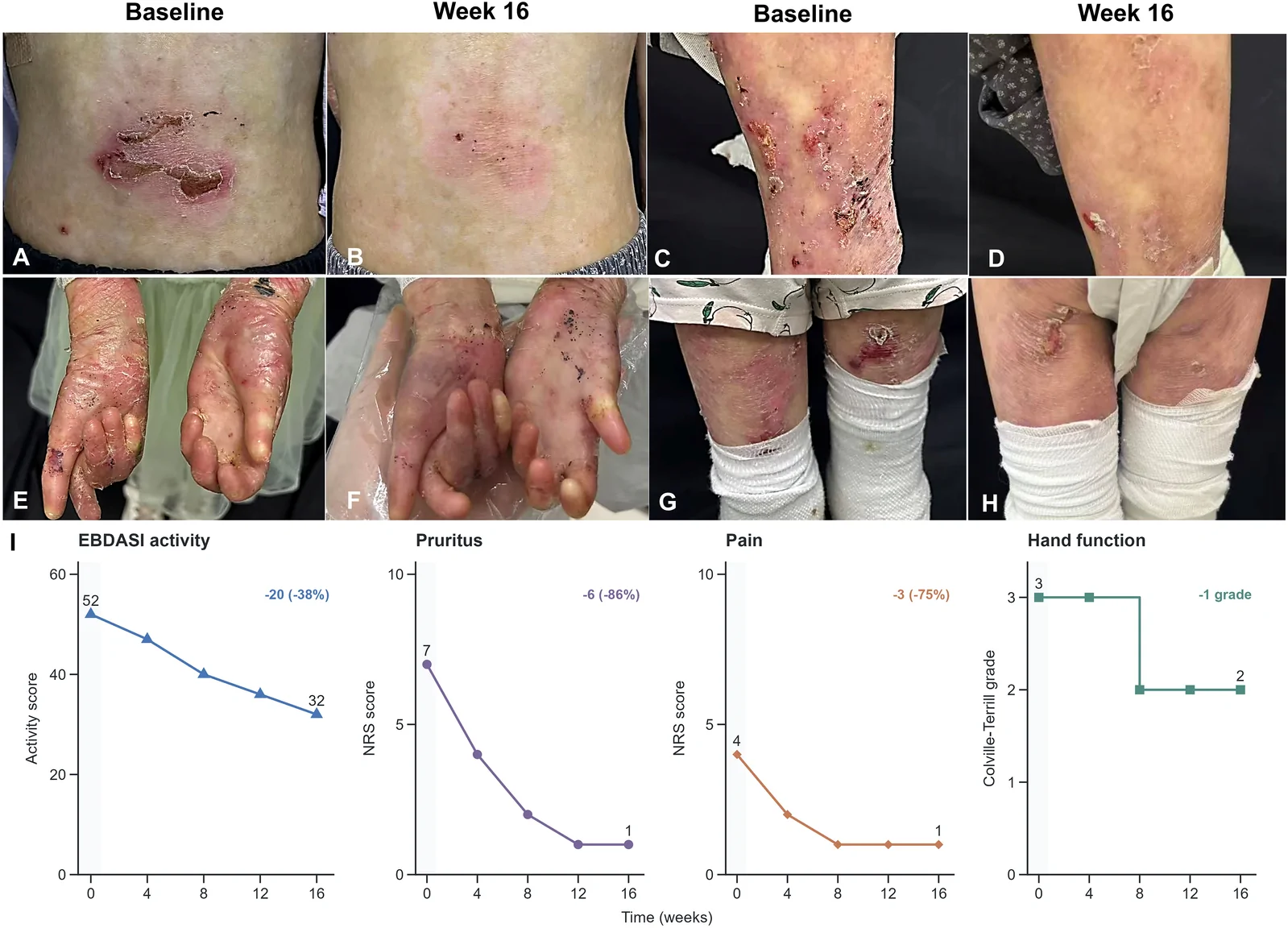
Clinical course during 16 weeks of dupilumab treatment. The 25-kg patient received a 600-mg loading dose followed by 300 mg every 4 weeks. Routine wound care continued; according to caregiver report, cetirizine drops and topical fusidic acid cream were used intermittently and irregularly, without a prospective medication or application log. **(A, B)** The truncal erosion re-epithelialized by week 16, with residual erythema and dyspigmentation. **(C, D)** The forearm showed less erythema, excoriation, erosion, and crusting. **(E, F)** The palms showed fewer inflammatory and eroded lesions, with persistent scarring and contractures. **(G, H)** The knees showed fewer erosions and crusted lesions. **(I)** Prospective assessments at weeks 0, 4, 8, 12, and 16 showed decreases in pruritus NRS from 7 to 1 (-86%), EBDASI activity score from 52 to 32 (-38%), and pain NRS from 4 to 1 (-75%); hand-function grade changed from 3 to 2. The same trained physician assessed EBDASI activity. The child and caregiver jointly reported 24-hour NRS values. Photographs were obtained under broadly comparable, but not strictly standardized, conditions. These uncontrolled changes occurred during continued background care and do not establish causality.

At week 16, the large truncal erosion had re-epithelialized, with residual erythema and post-inflammatory dyspigmentation. The forearm, palms, and knees showed fewer inflammatory erosions and crusted lesions, although scarring and digital contractures persisted (**Figures 3A–H**). At the time of manuscript preparation, follow-up was ongoing and dupilumab 300 mg every 4 weeks was continuing.

## Discussion

### Diagnostic and phenotypic interpretation

The clinical phenotype, subepidermal blister on routine histopathology, familial segregation, and two *COL7A1* variants in trans supported RDEB. The molecular interpretation nevertheless remains cautious. ClinVar contains discordant pathogenic and uncertain-significance submissions for p.Gly1869Arg (15), and no variant-specific functional assay was performed. For c.5532+5G>A, the splice and protein evidence was derived from an unrelated patient (17), not from patient-derived RNA or skin in this child. Thus, the present case directly supports phase and phenotype-genotype concordance, whereas variant-specific functional inference is literature-derived.

The phenotype is best described as RDEB with a prominent pruritic-inflammatory component rather than definitive RDEB pruriginosa. Generalized severe or intermediate RDEB typically begins with widespread mechanically induced blistering and wounding in infancy; scarring, mucosal and nail involvement, and contractures may dominate, although pruritus can be substantial (1, 3). RDEB pruriginosa instead features intense itch with hypertrophic lichenified papules, plaques, or nodules, often linear and concentrated over pretibial or extensor sites; intact blisters may be sparse in classic presentations (1, 3). Chronic scratching in any RDEB phenotype can produce excoriations, crusting, lichenification, and secondary prurigo-like thickening. In this child, blistering began in infancy and remained generalized, with mucosal and nail involvement, widespread erosions, scarring, and digital contractures. Typical pretibial hypertrophic prurigo-like nodules were not documented. These features argue against assigning the pruriginosa subtype solely on the basis of severe itch and scratching-related changes.

### Clinical response in context

The primary clinical observation was a decrease in pruritus NRS from 7 to 1 over 16 weeks. Secondary descriptive changes included reductions in the EBDASI activity score from 52 to 32 and pain NRS from 4 to 1, fewer family-reported new blisters, a change in hand-function grade from 3 to 2, and photographic improvement in erosions and crusting. The EBDASI change reflects disease activity rather than the EBDASI total or damage score. Persistent scarring and contractures indicate that structural damage was not reversed. Continued wound care and incompletely documented, caregiver-reported intermittent use of cetirizine and topical fusidic acid, together with variation in mechanical trauma and natural disease fluctuation, may have contributed. The temporal association therefore does not establish that dupilumab caused the observed changes.

Dupilumab would not be expected to correct the underlying type VII collagen deficiency. Its rationale concerns a proposed secondary neuroimmune amplification loop in which repeated barrier disruption sustains inflammation and pruritus, while scratching adds mechanical injury. Cohort-level RDEB immune profiling and experimental sensory-neuron studies provide biological plausibility for IL-4/IL-13 blockade (3–5). However, scattered tissue eosinophils were nonspecific, and no serum, cellular, or tissue type 2 biomarkers were measured. Clinical responsiveness is not a mechanistic biomarker and cannot establish an individual type 2 inflammatory endotype, pathway engagement, or restoration of type VII collagen.

Reduced itch is the most consistent signal in the published RDEB experience summarized in **Table 1**. Early reports mainly involved RDEB pruriginosa (6, 7, 18–21), whereas later reports included a 12-month-old child, individually identifiable RDEB cases in a mixed-epidermolysis-bullosa series, and retrospective cohorts containing severe, intermediate, and pruriginosa phenotypes (8–10, 22). These studies differ in age, genotype, phenotype, dose, outcome measures, and follow-up; some Chinese publications may include overlapping participants, and publication bias cannot be excluded. Their case counts should not be summed as independent observations. The present case extends this literature by documenting a child whose dominant phenotype was generalized blistering and wounding rather than classic pretibial pruriginosa, with prospective assessment of pruritus, EBDASI activity, pain, hand function, and photographs.

The every-4-week regimen was extrapolated from current US weight-tiered pediatric atopic dermatitis dosing (23). Every-2-week schedules in earlier RDEB reports reflect heterogeneous case-based experience and do not define an optimal interval. No validated RDEB-specific dose, treatment duration, or stopping rule exists. No adverse events considered related to dupilumab were observed over 16 weeks, but this duration cannot establish long-term safety.

### Limitations and implications

Strengths include mutually supportive clinical, histological, segregation, and variant-interpretation data, together with five prospective multidomain assessments. The same trained physician assessed EBDASI activity at each visit, but the unblinded single-investigator design remains susceptible to observer bias. Joint child-caregiver NRS reporting was subjective, the Colville-Terrill grade was coarse, and photography was broadly comparable rather than strictly standardized. Blister number and wound area were not prospectively quantified, and no validated sleep, EB-specific quality-of-life, or pediatric dermatology quality-of-life instrument was used.

This was a single uncontrolled case with 16 weeks of follow-up and continued wound care. Concomitant cetirizine and topical fusidic acid use was caregiver-reported, intermittent, irregular, and not prospectively recorded in sufficient detail to quantify exposure or changes over time. Topical fusidic acid therefore remains a potential confounder for changes in crusting, exudation, and inflammatory wound appearance, and the appropriateness of its use from an antimicrobial stewardship perspective cannot be assessed from the available data. Natural fluctuation, regression to the mean, differences in mechanical trauma, and observer or caregiver expectations may have influenced the outcomes. Systematic hematological, biochemical, anthropometric, and nutritional assessments were unavailable, so an effect of unmeasured nutritional factors on wound healing cannot be excluded. Immunofluorescence mapping, electron microscopy, patient RNA analysis, variant functional studies, and type 2 biomarkers were also unavailable. The observations therefore do not establish efficacy attributable to dupilumab or a type 2 inflammatory endotype. Prospective multicenter studies should prespecify pruritus as a primary outcome and assess disease activity, pain, blistering, wound area, hand function, and mechanistic biomarkers. The present case supports further evaluation of dupilumab as a symptom-modifying adjunct for severe pruritus in selected patients with RDEB.

## Patient perspective

The patient’s family stated, “We understand that there is currently no cure for her condition. Since she began dupilumab treatment, however, her itching has improved markedly, and she can now sleep through the night. She has also developed far fewer new blisters. We feel that her overall daily well-being has also improved.

## Data Availability

The original contributions presented in the study are included in the article; further inquiries can be directed to the corresponding authors.
